# Supplementary material for: The MO25 protein Pmo25 functions in contractile ring stability and Sid2 localization during cytokinesis
Source: iScience. 2025 Nov 28;29(1):114287. doi: 10.1016/j.isci.2025.114287 (PMC12803953; doi:10.1016/j.isci.2025.114287)
Supplement: Document S1. Figures S1–S5 and Table S1 [file mmc1.pdf]

iScience, Volume *29*

Supplemental information

The MO25 protein Pmo25 functions in contractile ring stability and Sid2 localization during cytokinesis

Yanfang Ye, Sha Zhang, Jack R. Gregory, Aysha H. Osmani, Evelyn G. Goodyear, Davinder Singh, and Jian-Qiu Wu

Table S1. *S. pombe* strains used in this study. Related to the STAR methods.

Strain name	Genotype	Figure/Table/Movie/ Reference
JW8614	*wsc1-mEGFP-kanMX6 rlc1-mCherry-natMX6 sad1-mCherry-natMX6 ade6 ura4 leu1-32 his5Δ?*	Figure 1
JW8615	*pmo25-21-2-his5*$^{+}$*-kanMX6 wsc1-mEGFP-kanMX6 rlc1-mCherry-natMX6 sad1-mCherry-natMX6 ade6 ura4 leu1-32 his5Δ?*	Figure 1
JW3186	*h*$^{-}$ *kan*^{s}*-mYFP-4Gly-cdc15 Patb2-CFP-atb2 ade6 leu1-32*	Figure 1
JW8148	*pmo25-2 kan*^{s}*-mYFP-4Gly-cdc15 Patb2-CFP-atb2 ade6 leu1-32*	Figure 1, Movie 1
JW8333	*orb6-25 kan*^{s}*-mYFP-4Gly-cdc15 kan*^{s}*-Pmyo2-mCFP-myo2 Patb2-CFP-atb2 ade6 leu1-32*	Figure 1, Movie 1
JW2766	*bgs1-191 wsc1-mEGFP-kanMX6 rlc1-mCherry-natMX6 ade6-M210 leu1-32 ura4-D18 lys1*$^{+}$	Figure 1, Movie 2
JW8575	*pmo25-2 bgs1-191 wsc1-mEGFP-kanMX6 rlc1-mCherry-natMX6 ade6 leu1-32 ura4-D18 lys1*$^{+}$	Figure 1, Movie 3
	h^{+}*/h*$^{-}$ *pmo25*$^{+}$*/pmo25Δ::hphMX6 rlc1-tdTomato-natMX6/rlc1-tdTomato-natMX6 leu1*$^{+}$*::GFP-psy1/ leu1*$^{+}$*::GFP-psy1 Patb2-mRFP-atb2/Patb2-mRFP-atb2 ade6-M210/ade6-M216 ura4/ura4*	Figure 2; Movies 4 and 5
JW910	*h*$^{+}$ *kanMX6-Pcdc4-mYFP-4gly-cdc4 ade6-M210 leu1-32 ura4-D18*	Figure 3
JW8662	*pmo25-13Myc-hphMX6 ade6-M210 leu1-32 ura4-D18*	Figure 3
JW9877	*pmo25-13Myc-hphMX6 kanMX6-Pcdc4-mYFP-4Gly-cdc4 ade6-M210 leu1-32 ura4-D18*	Figure 3
JW10097	*kanMX6-Pcdc4-mYFP-4gly-cdc4 ade6-M210 ura4-D18*	Figure 3
JW10098-2	*pmo25-35::ura4*$^{+}$ *kanMX6-Pcdc4-mYFP-4gly-cdc4 ade6-M210 leu1-32 ura4-D18*	Figure 3
JW10070	*Patb2-CFP-atb2 cdc7-YFP-kanMX6 ade6 leu1-32*	Figure 4
JW10139	*sid2-mECitrine-kanMX6 ade6 ura4-D18 leu1-32*	Figure 4; Movie 6
JW10063	*pmo25-35::ura4*$^{+}$ *Patb2-CFP-atb2 cdc7-YFP-kanMX6 ade6 leu1-32 ura4-D18*	Figure 4
JW10138	*pmo25-35::ura4*$^{+}$ *sid2-mECitrine-kanMX6 ade6 ura4-D18 leu1-32*	Figure 4; Movie 7
YDM514	*h*$^{+}$ *sid2-13Myc-kanMX6 ade6-M21X leu1-32 ura4-D18*	Figure 4/Sparks et al.[26]
JW7943	*h*$^{-}$ *pmo25-mECitrine-kanMX6 ade6-M210 leu1-32 ura4-D18*	Figure 4
JW10082	*sid2-13Myc-kan pmo25-mECitrine-kanMX6 ade6 leu1-32 ura4-D18*	Figure 4
JW10302	*pmo25-tdTomato-natMX6 sid2-mEGFP-hphMX6 ade6 leu1-32 ura4-D18*	Figure 5; Movie 8
JW6810	*rlc1-tdTomato-natMX6 ags1Δ 3'UTR*$_{ags1}$$^{+}$*::ags1*$^{+}$*-GFP:leu1*$^{+}$*:ura4*$^{+}$*ade6 leu1-32 ura4-D18*	Figure 6
JW8677	*pmo25-21-2-his5*$^{+}$*-kanMX6 rlc1-tdTomato-natMX6 ags1Δ 3'UTR*$_{ags1}$$^{+}$*::ags1*$^{+}$*-GFP:leu1*$^{+}$*:ura4*$^{+}$ *ade6 leu1-32 ura4-D18*	Figure 6
JW5249	*GFP-bgs1-leu1*$^{+}$ *bgs1Δ::ura4*$^{+}$ *rlc1-tdTomato-natMX6 ade6-M210 leu1-32 ura4-D18*	Figure 6
JW8567	*pmo25-21-2-his5*$^{+}$*-kanMX6 GFP-bgs1-leu1*$^{+}$ *bgs1Δ::ura4*$^{+}$ *rlc1-tdTomato-natMX6 ade6 ura4 his5Δ?*	Figure 6
JW6153	*h*$^{-}$ *bgs4Δ::ura4*$^{+}$ *Pbgs4*$^{+}$*::GFP-bgs4*$^{+}$*-leu1*$^{+}$ *rlc1-tdTomato-natMX6 leu1-32 ura4-D18 his3-D1? ade6?*	Figure 6
JW8574	*pmo25-21-2-his5*$^{+}$*-kanMX6 bgs4Δ::ura4*$^{+}$ *Pbgs4*$^{+}$*::GFP-bgs4*$^{+}$*-leu1*$^{+}$ *rlc1-tdTomato-natMX6 leu1-32 ura4 his3-D1? ade6 his5Δ?*	Figure 6
JW8489	*eng1-mNeonGreen-kanMX6 ade6-210 ura4-D18 leu1-32 his5Δ?*	Figure 7

JW8488	*pmo25-21-2-his5⁺-kanMX6 eng1-mNeonGreen-kanMX6 ade6-210 ura4 leu1-32 his5Δ?*	Figure 7
JW6655	*h⁺ ync13-13Myc-natMX6 ade6-210 leu1-32 ura4-D18*	Figure 8
JW7943	*h⁻ pmo25-mECitrine-kanMX6 ade6-M210 leu1-32 ura4-D18*	Figure 8
JW7973	*pmo25-mECitrine-kanMX6 ync13-13Myc-natMX6 ade6-M210 leu1-32 ura4-D18*	Figure 8
JW7970	*pmo25-tdTomato-natMX6 ync13-mEGFP-kanMX6 ade6-M210 leu1-32 ura4-D18*	Figure 8
JW7968	*h⁺ sad1-mCherry-natMX6 pmo25-mECitrine-kanMX6 ade6-M210 leu1-32 ura4-D18*	Figure 9
JW5814	*sad1-mCherry-natMX6 ync13-mECitrine-kanMX6 ade6-M210 leu1-32 ura4-D18*	Figure 9
JW8174	*pmo25-mECitrine-kanMX6 kanˢ-Pmyo2-mCFP-myo2 Patb2-CFP-atb2 ade6 leu1-32 ura4-D18*	Figure 9
JW8135	*ync13-mECitrine-kanMX6 kanˢ-Pmyo2-mCFP-myo2 ade6 leu1-32 ura4-D18?*	Figure 9
JW8396	*ync13Δ::kanMX6 pmo25-mECitrine-kanMX6 kanˢ-Pmyo2-mCFP-myo2 Patb2-CFP-atb2 ade6 leu1-32 ura4-D18*	Figure 9
JW8136	*pmo25-2 ync13-mECitrine-kanMX6 kanˢ-Pmyo2-mCFP-myo2 ade6 leu1-32 ura4-D18?*	Figure 9
JW7964	*ync13-19-his5⁺-kanMX6 pmo25-mECitrine-kanMX6 ade6-M210 leu1-32 ura4*	Figure 9
JW7943	*h⁻ pmo25-mECitrine-kanMX6 ade6-M210 leu1-32 ura4-D18*	Figure 9
JW8137	*pmo25-2 ync13-mECitrine-kanMX6 Patb2-CFP-atb2 ade6 leu1-32 ura4-D18?*	Figure 9
JW8114	*ync13-mECitrine-kanMX6 Patb2-CFP-atb2 ade6-M210? leu1-32 ura4-D18*	Figure 9
JW81	*h⁻ ade6-210 leu1-32 ura4-D18*	Figure S1
JW2766	*bgs1-191 wsc1-mEGFP-kanMX6 rlc1-mCherry-natMX6 ade6-M210 leu1-32 ura4-D18 lys1⁺*	Figure S1
JW8330	*pmo25-2 ade6 leu1-32 ura4-D18*	Figure S1
JW8575	*pmo25-2 bgs1-191 wsc1-mEGFP-kanMX6 rlc1-mCherry-natMX6 ade6 leu1-32 ura4-D18 lys1⁺*	Figure S1
JW864	*h⁻ rlc1Δ::kanMX6 his7-366 leu1-32 ura4-D18 ade6-M216*	Figure S2
JW8330	*pmo25-2 ade6 leu1-32 ura4-D18*	Figure S2
JW10001	*rlc1Δ::kanMX6 pmo25-2 ade6 leu1-32 ura4-D18 his7-366?*	Figure S2
JW2104	*h⁺ ura4⁺-Pcdc12-ΔNFH3(Δ1-503)-cdc12-3YFP-kanMX6 ade6-M210 leu1-32 ura4-D18*	Figure S2
JW10028	*pmo25-35::ura4⁺ leu1-32 ura4-D18*	Figure S2
JW9994	*ura4⁺-Pcdc12-ΔNFH3(Δ1-503)-cdc12-3YFP-kanMX6 pmo25-35::ura4⁺ade6? leu1-32? ura4-D18*	Figure S2
JW10136	*pmo25-35::ura4⁺ mob1-tdTomato-kanMX6 ade6 ura4-D18 leu1-32*	Figure S3
JW10137	*mob1-tdTomato-kanMX6 ade6 ura4-D18 leu1-32*	Figure S3
JW10302	*pmo25-tdTomato-natMX6 sid2-mEGFP-hphMX6 ade6 leu1-32 ura4-D18*	Figure S4
JW8034	*h⁻ pmo25-mScarlet-I-kanMX6 ade6-M210 leu1-32 ura4-D18*	Figure S5
JW8894	*natMX6-Ppmo25-pmo25(aa19-329)-mScarlet-I-kanMX6 ade6-M210 leu1-32 ura4-D18*	Figure S5
JW81	*h⁻ ade6-210 leu1-32 ura4-D18*	Table 1

JW8330	*pmo25-2 ade6 leu1-32 ura4-D18*	Table 1
JW8307	*pmo25-20-his5*$^{+}$*-kanMX6 ade6-M210 leu1-32 ura4*	Table 1
JW8435	*pmo25-21-2-his5*$^{+}$*-kanMX6 ade6-210 ura4 leu1-32 his5Δ?*	Table 1
JW10028	*pmo25-35::ura4*$^{+}$ *leu1-32 ura4-D18*	Table 1
JW864	*h*$^{-}$ *rlc1Δ::kanMX6 his7-366 leu1-32 ura4-D18 ade6-M216*	Table 1
JW10001	*rlc1Δ::kanMX6 pmo25-2 ade6 leu1-32 ura4-D18 his7-366?*	Table 1
JW10002	*rlc1Δ::kanMX6 pmo25-20-his5*$^{+}$*-kanMX6 ade6 leu1-32 ura4-D18 his7-366?*	Table 1
JW10000	*rlc1Δ::kanMX6 pmo25-21-2-his5*$^{+}$*-kanMX6 ade6 leu1-32 ura4-D18 his5Δ? his7-366?*	Table 1
JW10031	*pmo25-35::ura4*$^{+}$ *rlc1Δ::kanMX6 his7-366? leu1-32 ura4-D18 ade6-M216?*	Table 1
MBY925	*h*$^{-}$ *myo2-ΔIQ1ΔIQ2::ura4*$^{+}$ *leu1-32 ura4-D18*	Table 1/D'Souza V et al.[129]
JW9989	*myo2-ΔIQ1ΔIQ2::ura4*$^{+}$ *pmo25-2 leu1-32 ura4-D18 ade6?*	Table 1
MBY53	*h*$^{-}$ *myo2-S1 ade6-M21x ura4-D18 leu1-32*	Table 1/Balasubramanian et al.[130]
JW10009	*pmo25-2 myo2-S1 ade6 leu1-32 ura4-D18*	Table 1
JW10013	*pmo25-21-2-his5*$^{+}$*-kanMX6 myo2-S1 ade6 leu1-32 ura4-D18 his5Δ?*	Table 1
MBY54	*h*$^{-}$ *myo2-S2 ade6-M21x ura4-D18*	Table 1/ Balasubramanian et al.[130]
JW10011	*pmo25-2 myo2-S2 ade6 leu1-32 ura4-D18*	Table 1
JW10012	*pmo25-21-2-his5*$^{+}$*-kanMX6 myo2-S2 ade6 leu1-32 ura4-D18 his5Δ?*	Table 1
JW2252	*myo2-E1 ade6 leu1-32 ura4-D18*	Table 1
JW10010	*pmo25-2 myo2-E1 ade6 leu1-32 ura4-D18*	Table 1
JW2104	*h*$^{+}$ *ura4*$^{+}$*-Pcdc12-ΔNFH3(Δ1-503)-cdc12-3YFP-kanMX6 ade6-M210 leu1-32 ura4-D18*	Table 1
JW9993	*ura4*$^{+}$*-Pcdc12-ΔNFH3(Δ1-503)-cdc12-3YFP-kanMX6 pmo25-2 ade6 leu1-32 ura4-D18*	Table 1
JW9994	*ura4*$^{+}$*-Pcdc12-ΔNFH3(Δ1-503)-cdc12-3YFP-kanMX6 pmo25-35::ura4*$^{+}$*ade6? leu1-32? ura4-D18*	Table 1
JW2249	*rng2-346 ade6-M210 leu1-32 ura4-D18*	Table 1
JW10014	*pmo25-2 rng2-346 ade6 leu1-32 ura4-D18*	Table 1
JW994	*h*$^{-}$ *cdc4-8 his7-366 leu1-32 ura4-D18 ade6-M216*	Table 1
JW9986	*cdc4-8 pmo25-2 leu1-32 ura4-D18 ade6 his7-366?*	Table 1
JW10003	*cdc4-8 pmo25-21-2-his5*$^{+}$*-kanMX6 ade6 leu1-32 ura4-D18 his5Δ? his7-366?*	Table 1

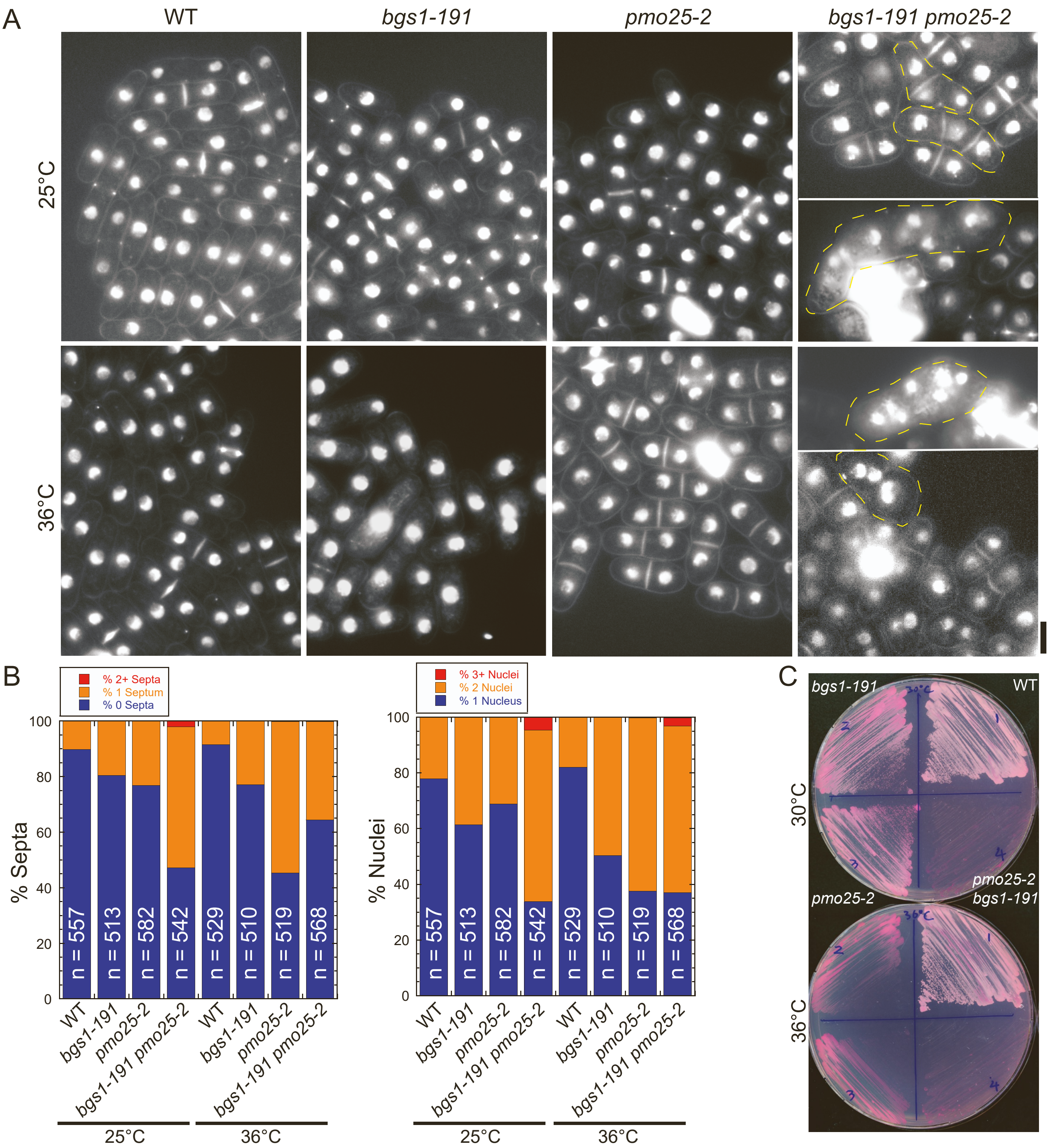

Figure S1. Synthetic genetic interaction between mutations in *pmo25* and the β-glucan synthase *bgs1/cps1*. Related to Figure 1. (A and B) Micrographs of middle focal plane (A) and quantification (B) of Hoechst 33258 stained cells that were grown in log phase at 25°C for ~41 h, then shifted to 36°C for 2 h before staining. Dashed lines mark cell boundary of multinucleated cells (not all the nuclei are visible in the quantified middle focal plane). In addition, lysed or shrunken multinucleated cells have very bright glowing staining, which is difficult to quantify so the nuclei are underestimated for the *bgs1-191 pmo25-2* strain. Bar, 5 µm. (C) Growth of the indicated strains (the same strains on both plates) on YE5S + Phloxin B (red dye accumulated in dead but not healthy cells) plates at 30 or 36°C for 38 h. The number of independent repeats: A-C = 1. The number of cells counted is directly shown in the figure graphs.

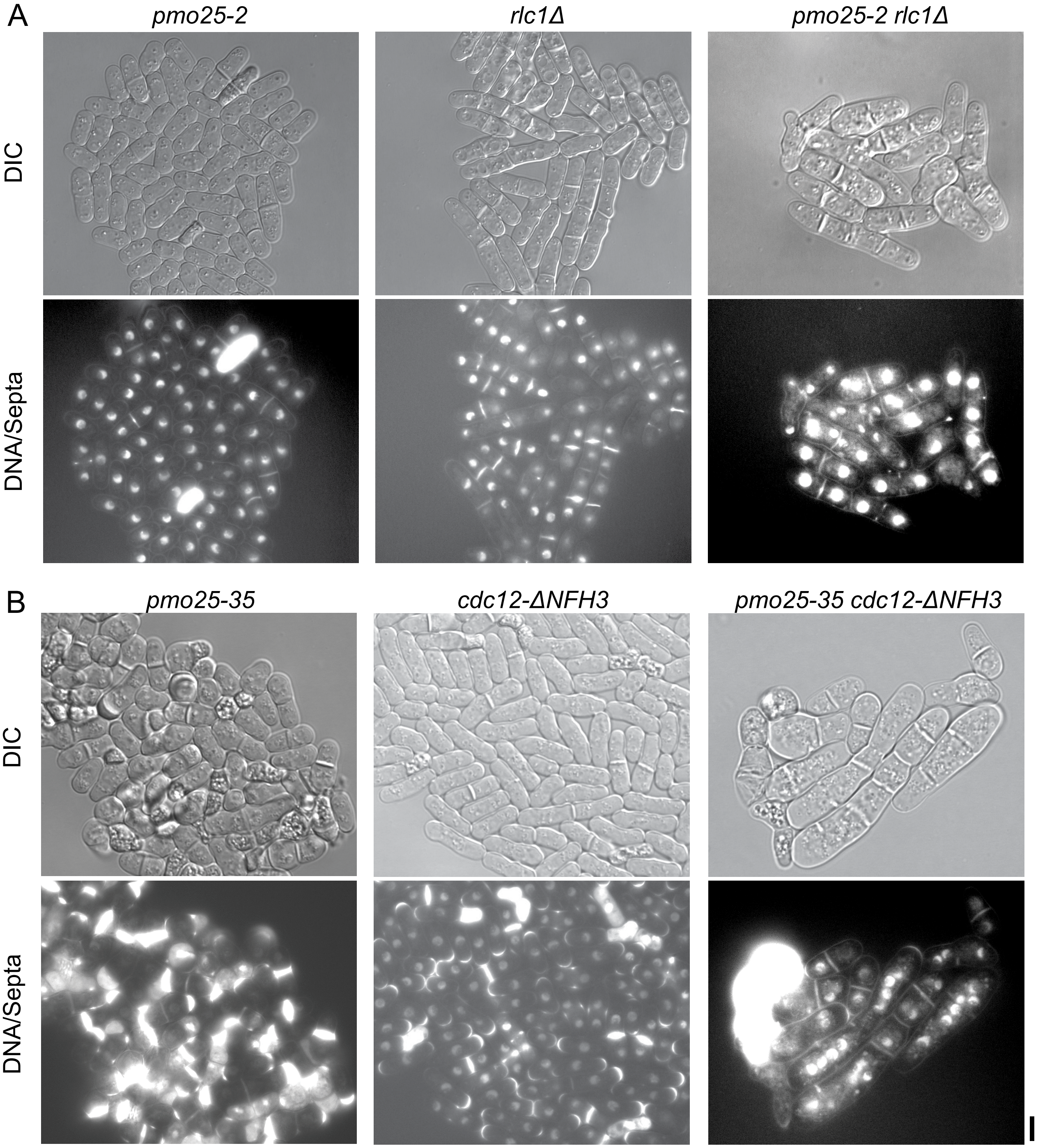

Figure S2. Synthetic genetic interactions between mutations in *pmo25* and the contractile-ring proteins myosin light chain Rlc1 (A) and formin Cdc12 (B). Related to Table 1. Images of DIC and DNA/Septa stained by the Hoechst 33258 (bisbenzimide) dye are shown. Lysed or died cells have the brightest glowing staining that obscures the neighboring cells.

(A) Cells were grown at 32°C then shifted to 25°C for 7 h before staining and imaging.

(B) Cells were grown at 25°C then shifted to 36°C for 6 h before staining and imaging.

The number of independent repeats: A = 2, B = 2. Bar, 5 μm.

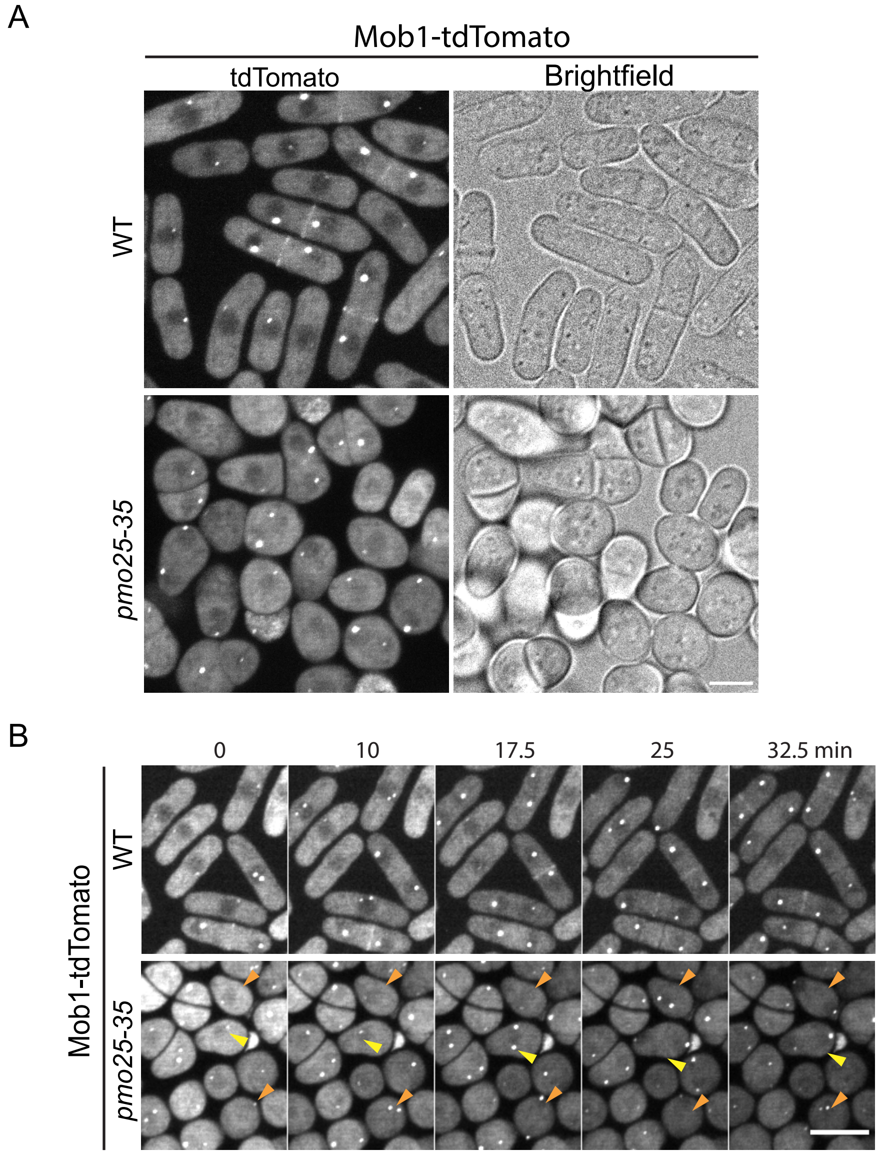

Figure S3. Pmo25 is involved in the recruitment of Mob1 to the division site. Related to Figure 4.

(A and B) Single time point images (A) and time course (in min) (B) of Mob1-tdTomato in WT (JW10137) and *pmo25-35* mutant (JW10136) grown at 36°C for 5 h before imaging at 36°C. Arrowheads mark the representative cells with weak or no Mob1 signal at the division site during cytokinesis. The number of independent repeats: A = 2, B = 2. Bars, 5 μm.

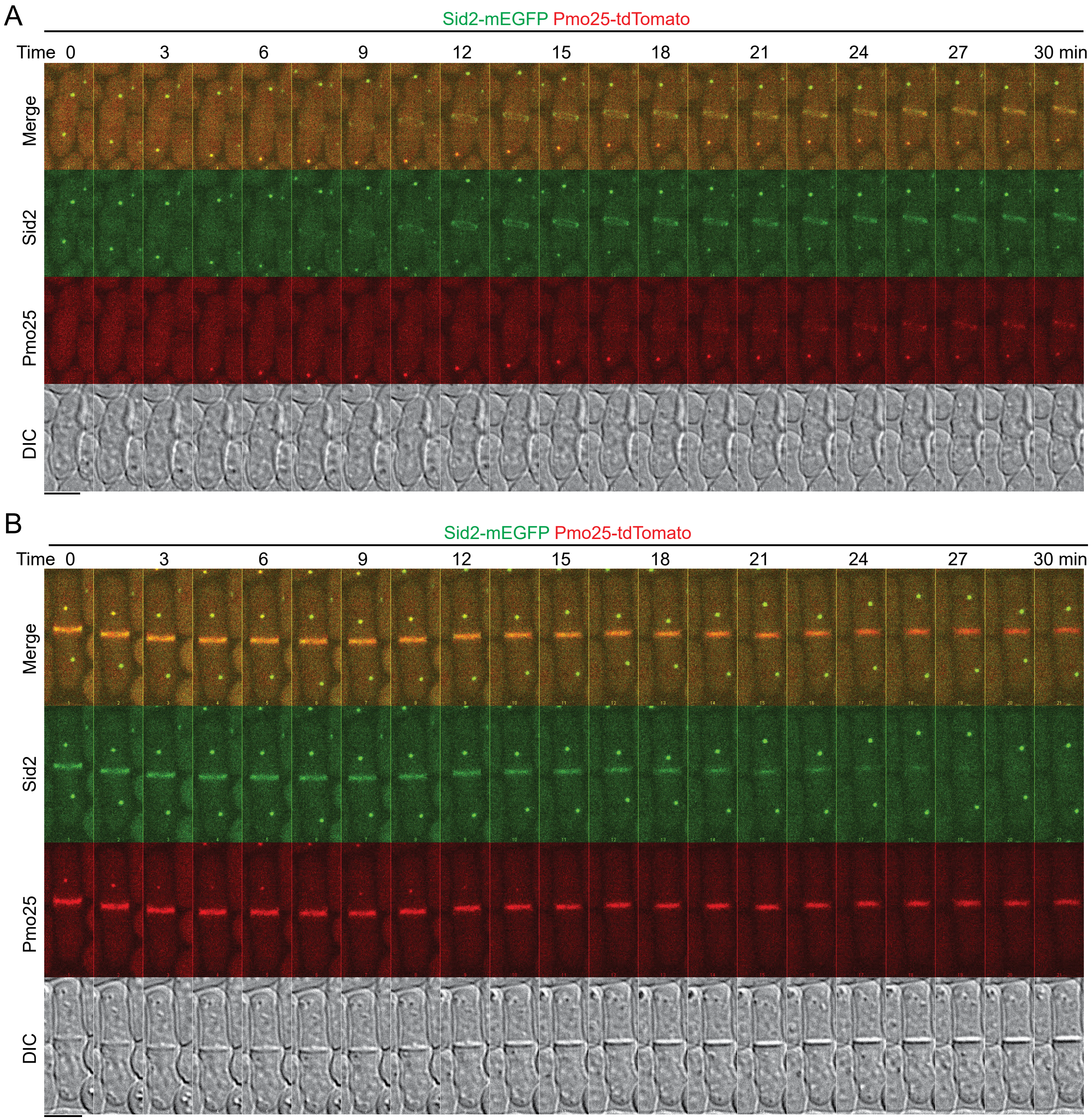

Figure S4. Time courses showing localizations of Sid2 and Pmo25 at the SPBs and the division site during cytokinesis. Related to Figure 5.
(A and B) Max intensity projections (9 slices with 0.8 μm spacing) and DIC images showing localizations of Sid2 and Pmo25 in two representative cells. Cells expressing both Sid2-mEGFP and Pmo25-tdTomato (JW10302) were imaged in time-lapse movies over 30 min. Cells were grown at 25°C for ~48 h before imaging.
The number of independent repeats: A = 2, B = 2. Bars, 5 μm.

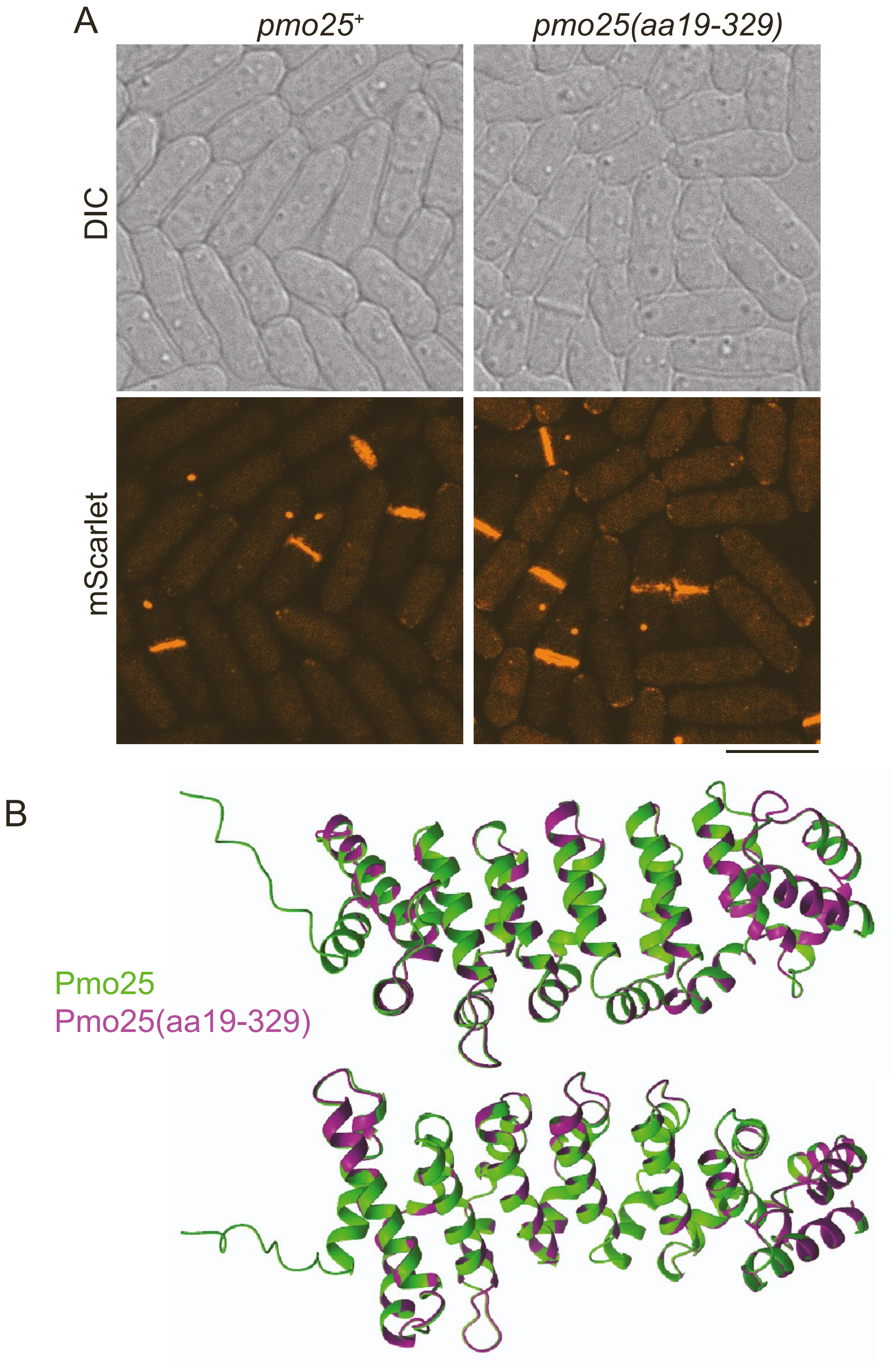

Figure S5. The morphology and localization of Pmo25-mScarlet and Pmo25(aa19-329)-mScarlet NH_2 terminal truncation mutant. Related to Figure 8.

(A) Images of cells expressing Pmo25-mScarlet (JW8034) or Pmo25(aa19-329)-mScarlet mutant (JW8894). mScarlet images are maximal intensity projection of 11 Z slices with 0.5 μm spacing. Bar, 5 μm.

(B) Two views (with ~90 °rotation) of the overlay of the predicted structures of full length Pmo25 (green) and Pmo25(aa19-329) (purple) by AlphaFold3. The models were aligned using the Matchmaker structure analysis tool on ChimeraX.

The number of independent repeats: A = 2, B = 3.
